# Retrograde intrarenal surgery in the prone split-leg position for female upper urinary tract stones: a preliminary study of 16 cases

**DOI:** 10.3389/fsurg.2025.1562484

**Published:** 2025-04-24

**Authors:** Yu Cao, Mingshi Li, Wanbin Cao, Li Tang, Shenglan Chen

**Affiliations:** Department of Urology, Guangji Hospital, Zhongshan, Guangdong, China

**Keywords:** female, prone split-leg position, retrograde intrarenal surgery, upper urinary tract stones, flexible ureteroscopy

## Abstract

**Objectives:**

This study aims to preliminarily investigate the potential technical advantages of the prone split-leg position for treating upper urinary tract stones and evaluate its efficacy using computed tomography (CT).

**Materials and methods:**

A retrospective analysis was conducted on the clinical data of 16 female patients who underwent retrograde intrarenal surgery (fURS) for upper urinary tract stones in the prone split-leg position at our hospital between July and September 2024. All patients were treated using flexible ureteroscopes and received CT scans before and after the operation to assess surgical outcomes.

**Results:**

All surgeries were successfully performed in the prone split-leg position, with an average operation time of 62.25 ± 25.92 min. The immediate stone clearance rate was 100%, and no complications were observed.

**Conclusion:**

Retrograde intrarenal surgery in the prone split-leg position is an effective treatment for upper urinary tract stones in female patients. This position facilitates ureteroscopic access to the renal pelvis and the insertion of ureteral guide sheaths, improves the immediate stone clearance rate, reduces intrarenal pressure, and represents an efficient, economical, and safe treatment method.

## 1 Introduction

Retrograde intrarenal surgery (fURS) is an effective treatment option for upper urinary tract stones. With the growing popularity of minimally invasive treatments and the use of high-power laser equipment, an increasing number of patients with upper urinary tract stones are opting for flexible ureteroscopic treatment (1). In recent years, the introduction of the tip-deflectable negative pressure suction ureteral guide sheath has provided a novel solution for managing larger stones, further optimizing fURS (2). Currently, flexible ureteroscopic lithotripsy (fURS) is typically performed in the supine lithotomy position. A comprehensive search of PubMed was conducted using precise search terms: “(retrograde intrarenal surgery) AND (prone position)” and “[flexible ureteroscopy (fURS)] AND (prone position)”. Through a systematic literature review, several reports were identified that documented the use of percutaneous nephrolithotomy combined with flexible ureteroscopy (fURS) in the prone position. Notably, however, no studies were found that explicitly described the exclusive use of flexible ureteroscopy (fURS) in the prone position for managing upper urinary tract stones. We clarify that our study was approved by the Institutional Ethics Committee of Zhongshan Guangji Hospital (Approval Number: GJ-LL006) and was conducted in full accordance with the principles of the Declaration of Helsinki. Prior to enrollment, all eligible adult patients (aged 18 years and older) received a comprehensive explanation of the potential benefits and risks associated with the surgical procedure. Following this explanation, written informed consent was explicitly obtained from each participant, and their signed consent forms were duly documented as part of the study records.

## 2 Materials and methods

### 2.1 Materials

This study included 16 female patients admitted to our hospital between July 2024 and September 2024 who underwent retrograde intrarenal surgery (fURS) in the prone split-leg position for the treatment of upper urinary tract stones. All surgeries were performed by the same surgeon, who had experience with over 3,000 fURS cases. The inclusion criteria were defined as female patients with upper urinary tract stones, including ureteral and renal pelvic stones, measuring greater than 1 cm in diameter. Conversely, the exclusion criteria comprised patients with stone diameters greater than 3 cm, individuals under 18 years of age, and those with a history of neuromuscular disease, congenital renal abnormalities, coagulopathy, morbid obesity, or skeletal deformities. Among the cases, six were completed in a single stage. Ten cases, however, involved preoperative indwelling of stent tubes for an average duration of 31 ± 5.40 days. The procedures utilized the ZebraScope™ single-use digital flexible ureteroscope, the Raykeen 60 W Holmium YAG Laser, and the Disposable Ureteral Guide Sheath for Single Use (manufactured by Jinan Zhongkangshun Medical Devices Co., Ltd.).

General patient information before the operation is summarized in Table 1.

**Table 1:** General preoperative conditions (*x* ± *s*).

Age	Side (*n*)		Maximum stone diameter	Preoperative catheterization (*n*)		Reason for catheterization (*n*)		Days of catheterization
(Years old)	Left	Right	mm	Catheterized	Not catheterized	Infection	Stenosis	(Days)
37.88 ± 8.94	8	8	16.50 ± 5.15	10	6	8	2	31 ± 5.40

### 2.2 Surgical techniques

**Figure 1:**
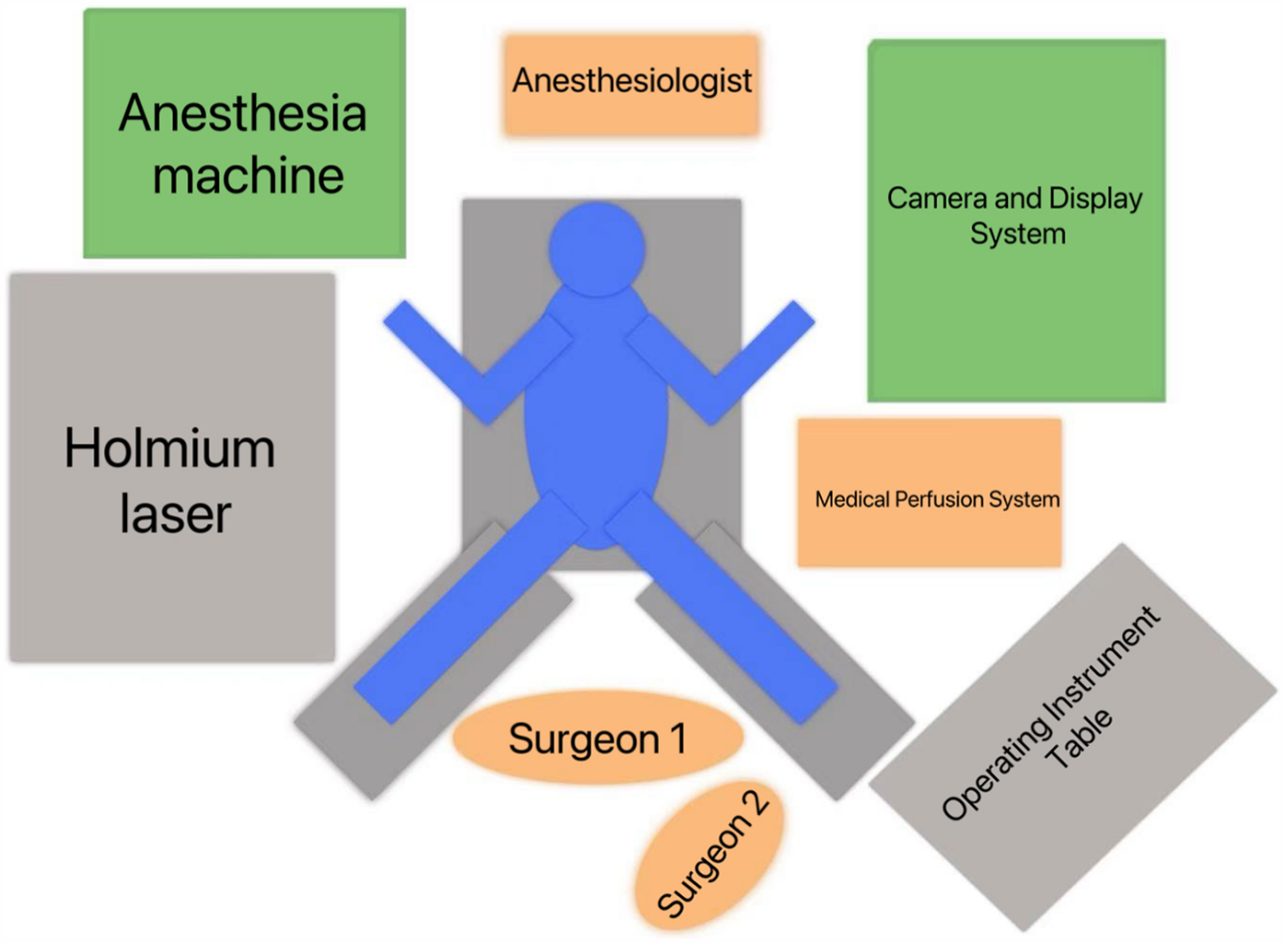
The schematic diagram shows the position of the patient in prone-split leg position, the position of the surgeon and the placement of the instruments.

After general anesthesia was administered, the patient was positioned in the prone split-leg position. The sacrococcygeal region extended approximately 5 cm beyond the edge of the operating table, and both lower limbs were abducted at an angle of 70–80 degrees and placed on the leg support boards (as shown in Figure 1). Six patients underwent transurethral exploration of the ureter using an 8/9.8 Fr rigid ureteroscope, while the remaining ten patients had their ureteral stents removed from the affected side before exploring the ureter up to the renal pelvis. For stones located in the upper ureter, the “billiard cue” technique (3) was employed to push the stones into the kidney. A pneumatic lithotripter was used to fragment the edges of the stones, which were then loosened and pushed into the kidney. Following exploration of the renal pelvis using the rigid ureteroscope, a guidewire was placed, and the length of the rigid ureteroscope exposed outside the body was measured and recorded before withdrawing the scope. A 12 F flexible ureteral guide sheath was selected, and the insertion depth was determined based on the previously measured exposed length. The ureteral guide sheath was placed under the guidance of the guidewire, and the inner core of the sheath was withdrawn after reaching the predetermined depth. Using a perfusion pump set at a pressure of 80 mmHg and a flow rate of 200–300 ml/min, a flexible ureteroscope was advanced through the ureteral guide sheath to locate the stone and perform holmium laser lithotripsy. The holmium laser was set at 30 W (energy: 1 J; frequency: 30 Hz), fragmenting the stones into 2–3 mm pieces, which were then flushed out with perfusion fluid. After confirming the removal of all intrarenal stones and checking for residual fragments, a guidewire was placed, and the scope and ureteral guide sheath were withdrawn under the monitoring of the flexible ureteroscope. A 5 Fr double-J stent and a urethral catheter were left in place, marking the end of the procedure. Two hours after the operation, routine blood tests and calcitonin levels were rechecked. The urethral catheter was removed 12 h after surgery, and routine blood tests, kidney-ureter-bladder (KUB) radiography, and urinary CT were performed on postoperative day 1. The perioperative conditions are summarized in Table 2.

**Table 2:** Perioperative conditions (*x* ± *s*).

Preoperative white blood cell count, postoperative 2 h white blood cell count, postoperative day 1 white blood cell count, postoperative 2 h procalcitonin, operation duration, urine culture (*n*)						
10^9^/L	10^9^/L	10^9^/L	ng/ml	min	Negative	Positive
7.55 ± 2.36	8.93 ± 2.19	8.45 ± 1.39	0.11 ± 0.02	62.25 ± 25.92	4	12

## 3 Results

In this group of 16 patients who underwent retrograde intrarenal surgery (fURS) in the prone split-leg position, the average operation time was 62.25 ± 25.92 min. No intraoperative injuries to the kidney or ureter were observed, and no significant bleeding occurred. Two hours postoperatively, the average white blood cell count was 8.93 ± 2.19, and on postoperative day 1, the average white blood cell count was 8.45 ± 1.39. A follow-up CT scan within 24 h postoperatively revealed an immediate stone clearance rate of 100% in the renal collecting system. No postoperative complications, such as fever, infection, loss of renal function on the affected side, or other systemic complications, were observed. Among the 16 patients, all complications were categorized as Clavien-Dindo grade I. Specifically, 13 patients experienced mild gross hematuria, and 7 of these patients additionally reported urinary pain (all VAS scores were below 3). No patients required specialized interventions such as analgesics; instead, all were advised to increase fluid intake. Notably, all symptoms resolved within 24 h postoperatively.All patients were able to get out of bed and eat six hours after surgery, were discharged 48 h postoperatively, and had their ureteral stents removed 15 days after surgery. Postoperative follow-up computed tomography (CT) scans performed at 2 and 6 months postoperatively revealed no radiographic evidence of hydronephrosis, ureteral dilation, or stone recurrence. The comparison table between the prone position group and the supine position group is shown in Table 3.

**Table 3:** Prone position group vs. supine position group comparison table.

Comparison metrics	Prone position group	Supine position group
Operative time		
- Mean time (min)	62.25 ± 25.92	60.15 ± 21.92
Perfusion data		
- Mean perfusion volume (ml/min)	250 ± 50	100 ± 20
- Perfusion pressure (mmHg)	80 ± 5	80 ± 5
Postoperative complication scores		
- Grade I complication rate	81.2%	75%
Stone distribution (number of cases)		
- Renal pelvis stones	6	6
- Upper ureteral stones	10	10
Laser parameters		
- Mean energy (J)	1.0	1.0
- Frequency (Hz)	30	30
- Fiber diameter (μm)	200	200

## 4 Discussion

With the continuous advancement of endourological techniques and equipment, retrograde intrarenal surgery (fURS) has gained increasing recognition as an effective treatment for upper urinary tract stones (4). A key limiting factor for fURS is the stone retention rate, particularly the immediate stone clearance rate. Most patients require time for postoperative stone fragments and powder to be excreted naturally (5). This study explored the potential of positional changes to improve surgical efficiency and immediate stone clearance rates.

A review of the literature revealed that some scholars have performed percutaneous nephrolithotomy combined with retrograde ureteropyeloscopy in a fully prone position to treat complex renal stones (6). By reviewing the urological CT scans taken in the prone position, it was found that the calyces, renal pelvis, ureter, and the level of the urethral orifice show a stepped descent. An attempt was made to explore whether the characteristic of the stepped descent of the urological lumens in the prone position of the human body can be utilized to improve the efficiency of flexible ureteroscopic lithotripsy and the immediate stone clearance rate. However, no reports of fURS in the fully prone split-leg position have been published. In our study, it was observed that during retrograde intrarenal surgery in the prone split-leg position, the ureter between the bladder and the ureteropelvic junction was straighter compared to the supine position, and the angle of the ureteropelvic junction was significantly smaller. This is consistent with the previous findings of anatomical studies on urinary tract lumens in the prone position (6).

There were no difficulties in removing the ureteral stent or performing ureteral exploration above the endoscopic field-of-view of the ureterovesical junction in the prone position. However, the flexible ureteropyeloscopy exploration procedure differed slightly after the successful placement of the ureteral guide sheath. In the supine position, after entering the kidney, the flexible ureteropyeloscope handle is pushed upward, and the lens bends downward into the lower calyx. In contrast, in the prone position, the handle is pressed downward, and the lens bends upward into the lower calyx. Experienced urological surgeons can adapt quickly to this modified technique.

In the treatment of female upper urinary tract stones using fURS in the prone split-leg position, perfusion fluid flowed out of the body more efficiently through the guiding sheath compared to the supine position. Even in the absence of negative pressure suction, achieving sufficient filling of the renal pelvis and collecting system remains difficult, with endoscopic visualization revealing collapse of the intrarenal collecting system. To maintain appropriate filling, the perfusion flow rate was increased to 200–300 ml/min. Analysis of Table 3 reveals that, with the exception of a statistically significant difference in irrigation flow rate between the prone and supine positions, no significant disparities were observed in operative time, irrigation pressure, postoperative complication scores, or laser parameters. It is believed that, in the prone position, the ureter and urethra lie below the water level of the renal pelvis, with the external urethral orifice at the lowest point of the urinary tract. The perfusion fluid flows efficiently from high to low due to gravity and siphonage effects (7), resulting in a low-pressure state within the kidney. Several studies have investigated the impact of patient positioning on intrarenal anatomy, highlighting the advantages of the prone position, including reduced intrapelvic pressure (mitigating the risk of fluid absorption) and improved accessibility for establishing ureteroscopic access (8). Furthermore, the publication “Endoscopic Combined Intrarenal Surgery in Galdakao-Modified Supine Valdivia Position: A New Standard for Percutaneous Nephrolithotomy?” (9) indirectly corroborates the influence of the prone position on intrarenal anatomical configurations.

Good perfusion and return flow enhance visibility, enabling the use of higher-energy lasers to improve lithotripsy efficiency while avoiding local high temperatures and reducing surgical time. After stone fragmentation in the prone position, the fragments concentrated in the renal pelvis due to gravity. If a fragmented stone fell into the lower calyx, it could be shifted to the renal pelvis by adopting the Trendelenburg position. In the prone position, the perfusion fluid flowed out of the body more easily through the sheath, eliminating the need to pulverize the stone completely using a laser. Stone fragments of 2–3 ml were easily flushed out with the perfusion fluid. In this study, the longest operation time (105 min) was due to the difficulty in fragmenting the stone that had fallen into the lower calyx. The laser fiber had trouble making effective contact with the stone, thus prolonging the operation. Subsequently, by changing to the Trendelenburg position, the stone spontaneously moved to the renal pelvis under the influence of the irrigation fluid, and was then quickly dealt with. This shows that the prone position may have certain local advantages in dealing with lower calyx stones.

Complications of fURS include acute urinary tract infections, systemic inflammatory response syndrome, and sepsis. Risk factors are related to stone size, perfusion pressure, and stent indwelling time (10). In this study, eight patients with acute urinary tract infections and positive urine cultures underwent secondary surgery after the placement of indwelling ureteral stents and treatment with sensitive antibiotics. Two hours postoperatively, routine blood tests, calcitonin levels, and various inflammatory markers were within the normal range, and the patients' body temperatures remained normal. At 24 h post-operation, urinary CT scans showed no perinephric effusion or hematoma, and the patients reported no lower back pain or swelling. These findings confirm that the intrarenal pressure during fURS in the prone split-leg position was relatively low. Additionally, none of the 16 patients in this study required the use of a net basket, which also offers advantages in terms of conserving medical resources. Therefore, fURS performed in the prone split-leg position facilitates rapid stone fragmentation and clearance, shortening surgical time and improving efficiency.

The anesthesia risk associated with the prone vs. supine position during endourological surgery remains a topic of debate. Some scholars argue that the supine position reduces anesthesia risk by minimizing the burden on the heart and lungs (11), while others have found that the prone and lateral positions positively impact patient oxygenation (12). The current consensus is that both prone and supine positions are safe in terms of anesthesia risk (13). In this study, patients were repositioned from the supine position on the transfer bed to the prone position on the operating table after anesthesia induction. After surgery, they were returned to the supine position on the transfer bed and transferred to the ward after recovering from anesthesia. No abnormalities in blood oxygen saturation or vital signs were observed before, during, or after anesthesia. Therefore, the prone split-leg position for fURS does not compromise anesthesia safety.

The results of this study demonstrate that fURS performed in the prone split-leg position for the treatment of upper urinary tract stones in women achieves a high immediate stone clearance rate, making it a safe and effective treatment method. The stepped - descent of the urological lumens in the prone position may facilitate the flow of perfusion fluid. The action of gravity helps to ensure a more efficient flow of the fluid used during flexible ureteroscopic lithotripsy. This better - flowing perfusion fluid contributes to maintaining a clear visual field within the urinary tract, enabling urologists to perform lithotripsy more effectively. For example, it can flush away debris and blood more easily, preventing them from obscuring the view of the stone. The action of gravity on the stepped - descent structure may assist in the movement of stone fragments. Stone fragments generated during lithotripsy are more likely to move towards the urethral orifice, making them easier to expel. In contrast, in other positions, the fragments may be more likely to remain trapped in the calyces or other parts of the urinary tract, resulting in a lower immediate stone - clearance rate. Effectively utilizing this characteristic has the potential to revolutionize the way flexible ureteroscopic lithotripsy is performed. As the efficiency of both lithotripsy and stone - clearance is improved, the operation time may be shortened. This is beneficial to patients, reducing their anesthesia time and associated risks, and providing experience for subsequent treatment of upper urinary tract stones with a greater burden. A higher immediate stone - clearance rate means a lower risk of residual stone fragments, which can cause complications such as recurrent pain, obstruction, and infection. Therefore, by leveraging this anatomical feature, patients' prognosis may be significantly improved both in terms of short - term recovery and long - term prevention of urological problems related to retained stones.

This study demonstrates that performing flexible ureteroscopic lithotripsy (fURS) in the prone split-leg position for treating upper urinary tract stones in female patients achieves a high immediate stone clearance rate and is both safe and effective. The stepped urinary tract structure in the prone position may optimize the flow of irrigating fluid and the expulsion of stone fragments through gravitational forces, thereby reducing the risk of residual stones. Our research has several limitations. All enrolled patients were female, the stone burden was relatively low, and the study had a small sample size with a retrospective design. The strict and potentially biased selection criteria—such as including only female patients—were established to prioritize surgical safety. However, this may have introduced bias into the complication data. Further prospective studies with larger sample sizes are currently underway to confirm the safety and effectiveness of fURS in the prone split-leg position. Nonetheless, our preliminary findings have validated the feasibility of this approach and provided initial insights into patient selection, surgical techniques, and complication prevention. Looking forward, it is essential to increase the number of cases, include patients with heavier stone burdens, and compare differences between the prone and supine positions through pressure monitoring of the flexible ureteroscope. Subsequent efforts should also expand the sample size, evaluate the therapeutic outcomes of different positions, and explore its application in male patients, ultimately contributing valuable experience for managing high-burden stones.

## Data Availability

The original contributions presented in the study are included in the article/Supplementary Material, further inquiries can be directed to the corresponding author.
